# Methotrexate Ameliorates Systemic Inflammation and Septic Associated-Lung Damage in a Cecal Ligation and Puncture Septic Rat Model

**DOI:** 10.3390/ijms22179612

**Published:** 2021-09-04

**Authors:** Josep Bringué, Raquel Guillamat-Prats, Maria Luisa Martinez, Eva Torrents, Marta Camprubí-Rimblas, Lluís Blanch, Antonio Artigas

**Affiliations:** 1 Institut d’ Investigació i Innovació Parc Taulí (I3PT), 08201 Sabadell, Spain; bringuejosep@gmail.com (J.B.); mcamprubi@tauli.cat (M.C.-R.); lblanch@tauli.cat (L.B.); aartigas@tauli.cat (A.A.); 2 CIBER de Enfermedades Respiratorias (CIBERES), 08201 Sabadell, Spain; 3 Facultat de Medicina, Universitat Autonoma de Barcelona, 08193 Bellaterra, Spain; 4 Critical Care Center—Hospital Universitario General de Catalunya, 08190 Sant Cugat del Valles, Spain; makilu80@hotmail.com; 5 Critical Care Center—Corporació Sanitària i Universitària Parc Taulí, 08201 Sabadell, Spain; ralasa31@hotmail.com

**Keywords:** acute lung injury, sepsis, methotrexate, acute respiratory distress syndrome, systemic inflammation

## Abstract

Background: Sepsis is a serious, heterogeneous clinical entity produced by a severe and systemic host inflammatory response to infection. Methotrexate (MTX) is a folate-antagonist that induces the generation of adenosine and also inhibits JAK/STAT pathway; MTX it is widely used as an anti-inflammatory drug to control the immune system. Objective: The aim of this study was to assess the beneficial effects of a single and low dose of MTX in the systemic response and acute lung injury (ALI) induced by sepsis. As in the clinics, we treated our animals with antibiotics and fluids and performed the source control to mimic the current clinic treatment. Methods and main results: Sepsis was induced in rats by a cecal ligation puncture (CLP) procedure. Six hours after induction of sepsis, we proceeded to the source control; fluids and antibiotics were administered at 6 h and 24 h after CLP. MTX (2.5 mg/Kg) was administered 6 h after the first surgery in one CLP experimental group and to one Sham group. A protective effect of MTX was observed through a significant reduction of pro-inflammatory cytokines and a decrease infiltration of inflammatory cells in the lung. In addition, we found a regulation in adenosine receptor A2aR and the metalloproteinases by MTX. Conclusion: A single, low dose of MTX attenuates sepsis lung-associated damage by decreasing pro-inflammatory response, infiltration of pro-inflammatory cells and avoiding defective tissue lung remodeling.

## 1. Introduction

Sepsis is a serious, heterogeneous clinical entity produced by a severe host systemic inflammatory response to infection. It has a high incidence, with more than 50 million cases every year with a high mortality rate near 30% [1,2,3,4]. Although improvements in the management of sepsis and the early adequate antibiotic therapy [5,6,7] have led to significant decreases in early mortality. At present, there are no specific therapies to treat sepsis and new therapeutic treatments need to be developed [8,9,10]. Lungs are one of the main organs affected during sepsis and nearly 50% of patients with sepsis also develop an acute respiratory distress syndrome (ARDS) [11].

At the molecular level, the massive release of the bacterial lipopolysaccharide (LPS) and its interaction with the innate immune cells through the binding to Toll-like-receptors (TLRs) combined with CD14 result in the induction of the NF-kB-pathway and the release of a large number of inflammatory mediators such as tumor necrosis factor alpha (TNF-α) and the cytokines interleukin 6 and 10 (IL-6 and IL-10) [12,13]. An excessive systemic inflammatory response can lead to extensive organ and tissue damage ending into shock, multiple organ failure and death, especially if not recognized early and treated promptly [8,9,14].

Specifically in the lung, the infection and inflammation cause damage to the alveolar epithelium and endothelium producing edema, hemorrhage, diffuse damage and infiltration of neutrophils and macrophages. After, alveolar epithelium remodeling and additional cell extravasation will be controlled by the release of matrix metalloproteinases (MMPs) and regulated by its inhibitors TIMPs [15,16]. At later stages of sepsis, the adaptive immune system starts being a key player and there is an increase of the number of circulating T-regulatory-cells (Tregs) that are associated with immune-suppression [17,18,19]; this state intensifies susceptibility to secondary infections [20,21] with associated increased late mortality in patients with septic shock [14,22]. Complete resolution of infection requires an accurate balance between pro-inflammatory and anti-inflammatory pathways, repairing epithelial-endothelial damage and an equilibrium between the different T-cell populations [22,23,24,25].

Multiple anti-inflammatory and immunomodulating agents have been studied to combat the different phases of sepsis [26]. Of note, methotrexate (MTX) is a folate-antagonist that induces the generation of adenosine, which has anti-inflammatory properties [27], decreases superoxide-anion [28] and adhesion-molecules [29]. It has been shown that MTX is an inhibitor of JAK/STAT pathway activity [30]. MTX is a widely used drug to treat immune-mediated inflammatory diseases such as rheumatoid arthritis and several autoimmune diseases [31,32,33] and in low doses, MTX is generally safe and well-tolerated [34,35]. Interestingly, adenosine has been shown to reduce lung inflammation in the setting of LPS challenge, reduce thrombosis and decreases the formation of neutrophil extracellular traps (NETs) by neutrophils [36,37], which are hallmarks of sepsis, ARDS and other lung pathologies such as COVID-19 [38].

Resuscitation, antibiotics and source control is not enough for the management of sepsis; our hypothesis is that a low, single and intraperitoneal dose of MTX could control the systemic inflammatory response reducing sepsis lung-associated damage. In this study, we aimed to test the beneficial effect of MTX on sepsis-induced acute lung injury (ALI) in an experimental model of sepsis in rats induced by cecal-ligation and puncture (CLP) [39,40].

## 2. Results

### 2.1. Survival and Body Weight

**Figure 1:**
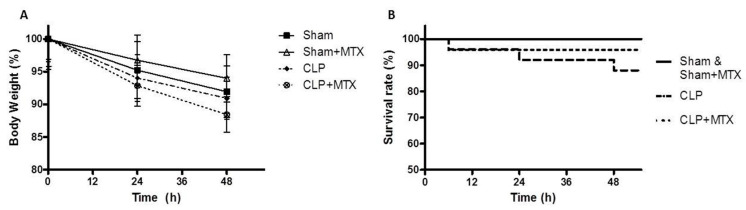
Body Weight and Survival. (**A**) Body weight measured at 0 h, 24 h and 48 h in all groups. (**B**) Representation of the survival rate in all groups in the curse of the experiment. Values represent group mean ± SEM. Sham and Sham + MTX *n* = 15, CLP and CLP + MTX *n* = 22.

All animals presented a loss of body weight due to the Sham or CLP surgery after 24 h and 48 h (Figure 1A). The CLPs groups presented a more striking body weight compared to the sham animals (Figure 1A), but any differences were significant. Over the course of the experiment, 12% of the septic group animals died while in the group of septic animals treated with MTX, the mortality was 4.2% and only before MTX administration. The survival rate between both septic groups, the non-treated with MTX and the treated one, was not statistically significant at 48 h (Figure 1B). All Sham and Sham + MTX rats survived to the end of the experiment (Figure 1B).

### 2.2. Systemic Inflammation

**Figure 2:**
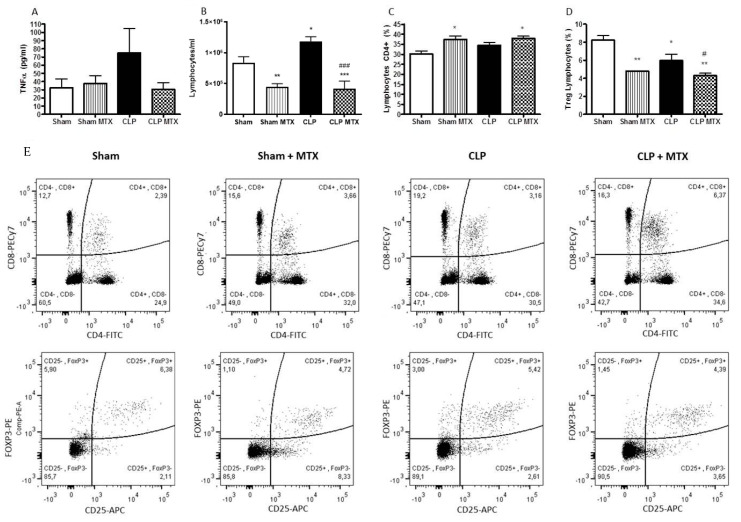
Systemic inflammation and blood lymphocytes activation. (**A**) Concentration of TNF-α in plasma measured by ELISA. (**B**) Number of total lymphocytes from blood cells. (**C**) Percentage of lymphocytes expressing CD4. (**D**) Proportion of CD4+ lymphocytes with a Treg phenotype (CD25+FoxP3+). (**E**) Representative flow cytometry dot plots of all groups. Gattering of CD4+ lymphocytes and Treg phenotype lymphocytes (CD25+FoxP3+). Data is expressed as mean ± SEM. Sham and Sham + MTX *n* = 4; CLP and CLP + MTX *n* = 6. * *p* < 0.05 vs. Sham group; # *p* < 0.05 vs. CLP group. ** *p* < 0.01. ***/### *p* < 0.001.

To assess the extent of systemic inflammation after MTX treatment, TNF-α concentration was measured in plasma 48 h after the surgery. TNF-α is an early pro-inflammatory cytokine produced at early stages of the diseases and keeps upregulated during some hours. After 48 h, we still observed an upregulated level of TNF-α in the CLP group, suggesting an enhanced pro-inflammatory state of this animals; the increase of TNF-α was completely absent with the MTX treatment (Figure 2A).

The number of total lymphocytes, CD4+ lymphocytes and Tregs (CD4+CD25+FOXP3+) were measured by FACS in blood. Septic rats (CLP group) presented an increase in the number of and lymphoid cells after 48 h; the MTX treatment decreased significantly the number of lymphoid populations, (in Septic and Sham groups) (Figure 2B). Regarding CD4+ T cells, MTX increased the proportion number of CD4+ cells in both treated groups. In septic animals, we observed a slight reduction in the proportion of cells in the Treg subpopulation compared to the sham group; however, a greater significant reduction in the number of Treg-cells was shown in the two groups treated with MTX; specifically in CLP + MTX group that presented a significant reduction compared to CLP group (Figure 2C,D). In Figure 2, we also show the representative dot plots for CD4+ and Tregs for each group.

### 2.3. Lung Weight and Bronchoalveolar Lavage Analysis

**Figure 3:**
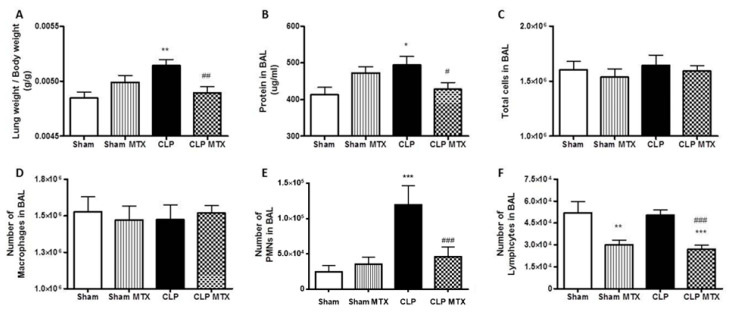
Lung weight and bronchoalveolar lavage analysis (BAL). (**A**) Lung weight corrected by the body weight. (**B**) Concentration of protein in the bronchoalveolar lavage. (**C**) Number of total cells in the bronchoalveolar lavage. (**D**) Number of total macrophages in the bronchoalveolar lavage (**E**) Number of total polymorphonuclear (PMNs) cells in the the bronchoalveolar lavage (**F**) Number of total lymphocytes in the the bronchoalveolar lavage. Data is expressed as mean ± SEM. Sham and Sham + MTX *n* = 13; CLP and CLP + MTX *n* = 18. * *p* < 0.05 vs. Sham group; # *p* < 0.05 vs. CLP group. **/## *p* < 0.01; ***/### *p* < 0.001.

Then, we studied the lung-associated damage produced by sepsis. The ratio lung weight/body weight was significantly increased in the CLP group, suggesting a damage into the lung. The MTX-treated CLP group presented a significant reduction in this ratio compared to CLP group (Figure 3A). We measured the total protein concentration in the BAL, a sign of lung permeability. The CLP group showed a significant increase compared to Sham and the treatment with MTX was able to reduce significantly the concentration of total protein in BAL suggesting a protected epithelial/endothelial barrier function (Figure 3B).

The influx of pro-inflammatory cells in the bronchoalveolar space is one of the main factors contributing to lung damage during sepsis. While MTX did not modify the total number of leukocytes or alveolar macrophages (Figure 3C,D), the MTX treatment in septic animals ameliorated polimorphonuclear-cells (PMN) infiltration in the bronchoalveolar space; the PMNs number was strongly amplified in the CLP group (Figure 3E). The number of lymphocytes in BAL was reduced by the treatment with MTX (Figure 3F), similarly as we observed in blood.

### 2.4. Inflammatory and Anti-Inflammatory Pathways in Lung

**Figure 4:**
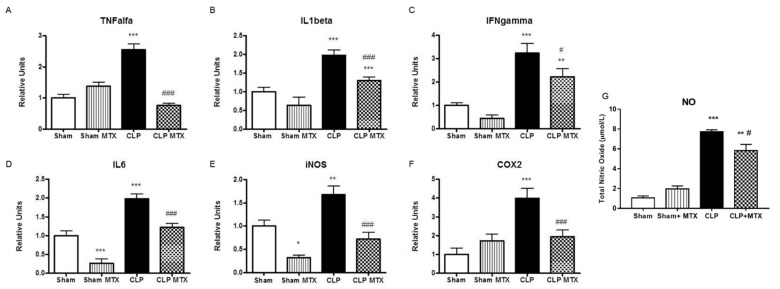
Gene expression of pro-inflammatory markers in lung tissue and total nitric oxide in bronchoalveolar lavage. (**A**–**F**): mRNA expression by qPCR. Data are expressed mean ± SEM, ∆Ct correction was applied using GAPDH as a housekeeping gene and units are relative to the expression of control group. Sham and Sham + MTX *n* = 10; CLP and CLP + MTX *n* = 15. (**G**) Nitric oxide measured in bronchoalveolar lavage. Sham and Sham + MTX *n* = 5; CLP and CLP + MTX *n* = 6. * *p* < 0.05 vs. Sham group; # *p* < 0.05 vs. CLP group. ** *p* < 0.01; ***/### *p* < 0.001.

The gene expression of several inflammatory cytokines was quantified in lung tissue. All the pro-inflammatory cytokines analyzed (IL-1β, IL6, TNF-α and IFNγ) were highly overexpressed in rat septic lungs and MTX administration significantly reduced the expression of these cytokines, clearly indicating an anti-inflammatory effect of this drug (Figure 4A–D). The expression of IL-6 and TNF-α in the CLP group treated with MTX reverted back to control levels. Moreover, sepsis resulted in a significant activation of the nitric oxide (NO) pathway by increasing the expression of the enzymes iNOS and COX2 (Figure 4E,F) and confirmed by the measure of total nitric oxide (NO) in BAL (Figure 4G). Compared with the Sham group, MTX significantly inhibited sepsis–induced production of NO, supported by the decrease in iNOS and COX2 mRNA expression and significant reduction in the released NO content in BAL (Figure 4E–G).

**Figure 5:**
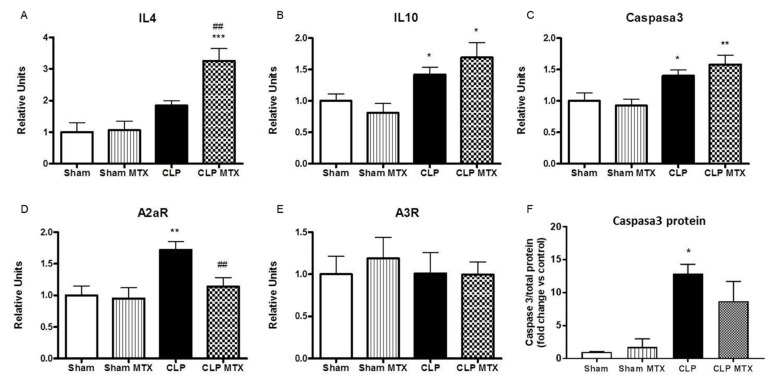
Gene expression of anti-inflammatory markers, adenosine receptors and caspase 3 and caspase 3 protein in lung tissue. (**A**–**E**): mRNA expression by qPCR and (**F**) Caspase3 protein in lung tissue. Data are expressed mean ± SEM, ∆Ct correction was applied using GAPDH as a housekeeping gene and units are relative to the expression of control group. Sham and Sham + MTX *n* = 10; CLP and CLP + MTX *n* = 15. * *p* < 0.05 vs. Sham group; ## *p* < 0.01 vs. CLP group. ** *p* < 0.01; *** *p* < 0.001.

The expression of the IL-4 and IL-10 anti-inflammatory cytokines was also measured. IL-4 expression was triggered by the treatment of the MTX in the CLP group (Figure 5A,B). In addition, an increase in the expression of IL-10 was observed in the septic groups, independently of the MTX treatment (Figure 5B). An increase in caspase-3 expression and protein synthesis suggests increased apoptosis in septic rat lungs, which was not reversed by MTX treatment (Figure 5C,F).

Furthermore, we determined the expression of adenosine receptors A2aR and A3R in lung tissue. Sepsis has been shown to increase the release of extracellular adenosine and increase the expression of adenosine receptors [41]. The expression of A2aR was increased in the CLP group and reduced by MTX treatment. No differences were observed in the levels of the A3R in any group (Figure 5D,E).

### 2.5. Recruitment Markers and Metalloproteinase Activation in Lung Tissue

**Figure 6:**
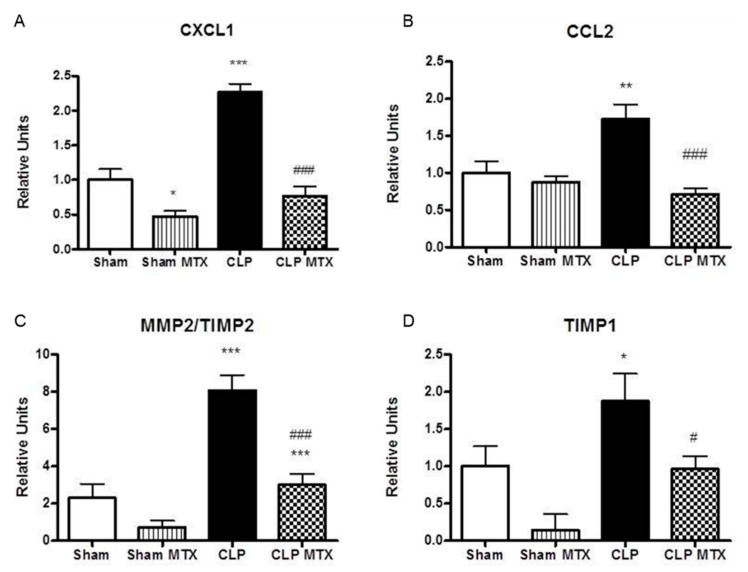
Gene expression of cell recruitment markers and metalloproteinases and its inhibitors in lung tissue. (**A**–**D**): mRNA expression by qPCR. Data are expressed mean ± SEM, ∆Ct correction was applied using GAPDH as a housekeeping gene and units are relative to the expression of control group. Sham and Sham + MTX *n* = 10; CLP and CLP + MTX *n* = 15. * *p* < 0.05 vs. Sham group; # *p* < 0.05 vs. CLP group. ** *p* < 0.01; ***/### *p* < 0.001.

The expression of CXCL1, a neutrophil recruitment marker and CCL2, a monocyte recruitment marker, were increased in septic rats. Interestingly, MTX significantly reduced the expression of CCL2 and CXCL1 expression to Sham levels after CLP (Figure 6A,B).

After damage, the lung parenchyma starts the recovering mechanism; at early stages of the acute lung damage is observed an increase of MMPs. The dysregulated increase of MMPs at early stages worsens extravasation and at later stages suggests a remodeling by fibrosis and is linked to a worse recovery prognosis. We observed an increase in the expression of the ratio of MMP2 to its inhibitor, TIMP2, increased in CLP rats, while MTX administration significantly reduced significantly the MMP2/TIMP2 ratio (Figure 6C,D). We measured TIMP1 as one of the main metallopeptidase inhibitors associated to lung fibrosis, TIMP1 expression augmented in the lung tissue after sepsis and it was successfully reduced in the CLP + MTX group (Figure 6D).

## 3. Discussion

This is the first preclinical study to analyze the effect of MTX in sepsis. In our study, we demonstrated that a single low dose of MTX had no observable negative effects such as increased mortality, loss of body weight or more systemic effects on septic animals. MTX treatment improved the systemic and lung innate immune responses, as evidenced by a reduction in the expression of several classical pro-inflammatory markers. In addition, we have shown an effect of MTX treatment in the adaptive immune response by a decrease of Tregs. MTX demonstrated a beneficial effect in acute lung damage by regulating also the later remodeling MMPs and TIMPs.

Sepsis is a high mortality disease which develops as a result of an overwhelming systemic inflammatory response to infection. In this study, CLP was performed to induce a polymicrobial sepsis with a mid-grade-severity in rats, a model that closely resembles the progression and characteristics of human sepsis [39,42,43]. Fluids and antibiotics were administered 6 and 24 h after the sepsis induction and the source of infection was removed 6 h after performing the CLP; the timing of the fluid and antibiotic therapy we used correlates to the initial care given when patients are admitted to the intensive care unit. Therefore, we chose to observe the animals 48 h after CLP, because they displayed a mild ALI at this point. The low mortality on our model suggests that the animals were developing a mild sepsis and did not show septic shock.

ARDS is a common complication during sepsis with high mortality rate. Our results show that MTX administration significantly reduced the number of neutrophils and lymphocytes recruited to the lung without altering the number of macrophages in the alveolus. MTX also reduced the expression of pro-inflammatory cytokines such as IL-1β, IL-6 and TNF-α, iNOS, IFN-γ and COX2, in the lung. The NF-κB-activation-pathway is regulated by LPS, generated during infection and the production of TNF-α and the activation of this pathway are central molecular events leading to the development of septic shock [44,45]. Systemically, MTX also reduced inflammation shown as a reduction of TNF-α. The inhibition of NF-κB-activation-pathway reduces the expression of multiple inflammatory genes, restores systemic hypotension and diminishes tissue neutrophil influx and microvascular endothelial barrier permeability [46]. Here, we have demonstrated that MTX decreases the expression of many pro-inflammatory cytokines, reduces NF-κB- pathway activation and significantly attenuates CLP–induced lung injury. Lipopolysaccaride and pro-inflammatory cytokines, such as TNF-α, iNOS, IFN-γ have been shown to induce iNOS in the endothelium and triggering the NO release [47]). Septic patients have shown to have elevated circulating and pulmonary nitrite/nitrate and NO products; the pathogenetic role of NO in sepsis comprises vascular damage in addition to the direct cellular toxicity of NO and NO-related compounds [48,49]. In murine experimental models, it has been shown an increase of the release of NO by the endothelium and enhanced iNOS enzyme activity [50,51]. Our treatment with MTX and the associated positive effects that we described are also supported by the decrease of total NO in BAL and iNOS expression in the lung tissue; other experimental models also described a beneficial effect in the reduction of iNOS and total NO linked to less epithelial and alveolar damage [52].Moreover, IL-4 expression is enhanced in the lung after MTX administration in septic rats. Type 2 cytokines, such as IL-4 and IL-13, trigger a specialized macrophage phenotype; more regulatory and anti-inflammatory macrophages, which is necessary for the adequate infection control [53,54,55].

Sepsis induces lymphocyte alterations in the number of circulating Treg-cells. High number of Tregs is associated with increased mortality in patients with septic shock [21,24]. We demonstrate that MTX reduced the number of Treg-cells and also slightly increased the total CD4+ population. It was reported that sepsis induces a decrease in CD4+ populations; therefore, it is important that any sepsis treatment not promote the depletion of this population, which is crucial to disease resolution [25,56]. The dysregulation of the immune system during sepsis produces a huge systemic inflammatory response in first phases and after immunosuppression which leads to death [17,18,19]. Consequently, control and maintenance of the CD4+ lymphocyte population is vital for the resolution of infection [57].

MTX is a folate antagonist that inhibits purine metabolism and RNA and DNA synthesis, leading to increased extracellular adenosine release at inflamed sites [58]. Adenosine interacts with A2AR which is expressed in lymphocytes and neutrophils [59,60]; the activation of A2AR is associated with an anti-inflammatory effect. A2aR activation reduces T cell function and proliferation [61]. Previously published data demonstrates that inhibiting A2AR reduces bacterial infection and mortality in septic patients [61,62]. Pre-clinical data with A2AR knock out mice models also had an increased survival rate, less pro-inflammatory response and limited systemic bacterial expansion than wildtype animals [63,64,65]. In our study, we observed that a single, low-dose of MTX decreased the expression of A2aR and all the described associated effects; furthermore, this change did not lead to an increase in the Treg population or to immunosuppression.

The recruitment of new pro-inflammatory cells to the lung tissue is also regulated by MTX. CCL2 and CXCL1 are involved in the recruitment of monocytes and neutrophils, respectively [66]. The recruitment of new pro-inflammatory cells is necessary for the infection resolution; however, a huge number of recruited inflammatory cells can trigger a worsening of the damage. To regulate the expression of recruitment markers may have a beneficial effect. Antagonism of CXCR2 or CCR2 (receptors for CXCL1 and CCL2 respectively) has been shown to dramatically reduce neutrophil recruitment and pulmonary inflammation and was previously used as a strategy to attenuate the septic process with significant therapeutic effects in endotoxemic animals [67,68]. Our results reveal that MTX reduces CXCL1 and CCL2 to control and protect against a worsen lung damage.

MMPs are activated in late stages of sepsis and stimulate extravasation and migration of leukocytes to the site of damage. MMP-2 has been shown to be involved in inflammation, recruitment of inflammatory cells, permeability and remodeling in lung and TIMP-1 has been studied as a prognostic factor for sepsis [29,34,35]. MTX was able to reduce the MMP2/TIMP2 ratio and the expression of TIMP-1 and we also observed a decrease in the total protein concentration in BAL, demonstrating a reduction in the permeability of the lung after MTX treatment. The reduction in the MMP2/TIMP2 ratio and TIMP-1 expression likely produced a beneficial effect in reducing lung injury in septic rats.

Our study design has some limitations. The CLP model cannot fully replicate all features of human disease and patients with sepsis usually present with other underlying diseases in addition that rats do not develop an immunosuppressive state at later stages of the disease. Nevertheless, our study using this experimental model is adding valuable information in how to use approved drugs to regulate the immune system in high mortality diseases. Pre-clinical experiments as the one we are here describing are necessary to further understand the basic mechanisms of sepsis development and resolution. We have focused only on lung injury produced by sepsis; however, the analysis of the effects of MTX on other organs could be interesting in future studies. We tested a unique and single dose of MTX, a more prolonged and continuous treatment could probably increase the beneficial effects.

The protective effect of MTX against ARDS could be attributed to the reduction of pro-inflammatory cytokines, a decline in infiltration of inflammatory cells in the lung and the polarization of lymphocytes to CD4+ that enhances the resolution of systemic infection.

In conclusion, our findings demonstrated that a single, low-dose MTX treatment prevents inflammation and immune-modulates the high systemic response to sepsis in a rat model. The administration of MTX in the pro-inflammatory phase of sepsis may be useful as a therapeutic agent targeting systemic inflammation and especially ARDS; our preclinical data support the use of MTX as a novel drug to modulate the inflammatory response in sepsis; however, these results need to be confirmed in the future in clinical studies.

## 4. Materials and Methods

### 4.1. Animal

Male Sprague–Dawley rats weighing 300–325 g at the beginning of the experiment were used, in accordance with the European Community Directive 86/609/EEC and Spanish guidelines for experimental animals. This study was approved by the institutional committee of “Universitat Autònoma de Barcelona” and the “Generalitat de Catalunya” (7812, 14/05/2014). The animals were randomly distributed into four experimental groups: Sham (15 animals/group), Sham + MTX (15 animals/group), CLP (22 animals/group) and CLP + MTX (22 animals/group).

### 4.2. Sepsis Induction and Treatment

Sepsis was induced by a cecal-ligation-puncture (CLP) procedure. CLP was performed under anesthesia with ketamine (100 mg/kg body-weight) and xylazine (10 mg/kg body-weight) administered by intraperitoneal injection. A laparotomy was performed, the cecum was identified and two ligatures were made with 3-0 silk sutures 1 cm and 3 cm from the end of the cecum. Three punctures were then performed with an 18G needle, after which the peritoneal cavity was sutured.

Six hours after induction of sepsis, we proceeded to the source control. The punctured part of the cecum was removed and the rest of the cecum was closed. Six and 24 h after CLP we performed an intradermal administration of saline (0.9% NaCl, 10 mL/kg), analgesic (buprenorphine 0.025 mg/kg; RB-Pharmaceuticals) and antibiotic (meropenem 20 mg/kg; Kern-Pharma, Lisboa, Portugal). In addition, one sham and one CLP group of animals were treated with one single dose of MTX (2.5 mg/kg; Pfizer, New York, NY, USA) or saline administered intraperitoneally 6 h after the first surgery. MTX dose was chosen by screening published pre-clinical studies that used MTX in acute models Forty-eight hours after sepsis induction, animals were sacrificed by exsanguination and blood samples, bronchoalveolar lavage (BAL) and lung tissue were collected. Samples were stored at −80 °C until analysis.

### 4.3. RNA Isolation and Gene Expression Analysis by qRT-PCR

Total RNA was extracted from lung tissue using TRIzol reagent (LifeTechnologies, Madrid, Spain). RNA was purified using chloroform, isopropanol and ethanol and quantified with Nanodrop-2000 (Thermo-Scientific, Wilmington, DE). Reverse transcription into cDNA was performed using the Reverse-Transcriptase-Core-kit to the manufacturer’s instructions (Eurogentec Seraing, Belgium). Quantitative real-time-PCR amplification was performed using SYBR- green (Eurogentec Seraing, Belgium) and the corresponding rat primers (Table 1). Data are expressed as target gene expression relative to GAPDH and compared with control group.

### 4.4. Western Blot of Caspase 3

For caspase-3 measurements, a piece of the right lung was homogenized in lysis buffer containing 1 mM sodium orthovanadate, protease inhibitor cocktail tablets (1 tablet for 250 mg of lung tissue) (Roche, Darmstadt, Germany), 0.5% Triton X-100, 150 mM NaCl, 15 mM Tris, 1 mM CaCl_2_ and 50 mM MgCl_2_ (pH 7.4) using a hand- held homogenizer. The homogenates were incubated for 30 min at 4 °C, centrifuged at 12,000 rpm at 4 °C for 20 min. The total protein concentration in lung homogenates was measured by the bicinchoninic acid method (Pierce; Thermo Scientific, Rockfort, IL). Equal amounts of proteins from lung homogenates were heat-denatured in Laemmli sample buffer with 2-mercaptoethanol (5%), resolved in 20% SD-PAGE gel and transferred to PVDF membranes (GE Healthcare, Little Chalfton, UK). Next, the blots were blocked with 5% PBS-non-fat dry milk for 2 h at room temperature (RT) and then incubated with mouse monoclonal anti-active caspase-3 (cleaved) primary detection antibody (1:1000) overnight at 4 °C. After thoroughly washing with 1 × PBS 0.05% Tween-20, the membranes were incubated for two hours at RT with a goat anti-mouse Texas Red as a secondary antibody. An anti-actin antibody was used as a loading control. Finally, the fluorescence signal was visualized and analyzed using an LAS4000 system (Fujifilm Life Science, Woodbridge, CT, USA).

### 4.5. Cellular Quantification of Bronchoalveolar Lavage, Cytokine and Total Nitric Oxide Measurement

BAL was undertaken by washing the right lung five times with 5 mL of saline via a tracheal cannula and it was centrifugated (500× *g*, 10 min, 4 °C). Supernatant was stored at −80 °C for protein measurement and pelleted cells were cytocentrifuged (Shandon-Scientific, Taper, Waltham, MA, USA) then allowed to dry in air for 15 min at RT. The cell yield and purity were assessed by Diff-Quick staining (Panreac, Castellar del Vallés, Spain).

The total protein concentration in BAL and plasma was quantified using a BCA protein assay kit (Thermo-Fisher, Rockfort, IL, USA) following the manufacturer’s protocol. The concentration of TNF-α in plasma was determined by multiplex assay (Luminex, Affymetrix, Darmstadt, Gemany).

Total nitric oxide was quantified with a colorimetric assay by using the Total Nitric Oxide and Nitrate/Nitrite Assay kit (R&D, Minneapolis, MN) and following the protocol provided by the manufacturer.

### 4.6. Flow Cytometry Analysis

For analysis of blood lymphocytes, blood samples were collected into tubes containing sodium citrate and surface–anti-rat -labelled antibodies: APC-CD3, FITC–CD4, PE-Cy5–CD25 and PerCP-eFluor710-CD8 antibodies followed by fixation, permeabilization and intracellular staining with PE-conjugated anti-rat Foxp3 antibodies (Figure 2). All antibodies were provided by Affymetrix, eBioscience. Controls were performed to enable correct compensation and confirm antibody specificity. After washing procedures, the stained cells were analyzed by flow cytometry (BD-FACSCanto, San Jose, CA). The results were analyzed with FlowJo software. Total T cells were gated by CD3+ cells, then CD3+CD4+ for CD4-T-lymphocytes and Tregs were gated by using CD3+CD4+CD25+FOXP3+.

### 4.7. Statistical Analysis

Data are expressed as mean ± SEM. The data were standardized and then parametric statistical tests were applied. One-way-ANOVA followed by Newman-Keuls-post-test was performed (GraphPad-Software Inc., La Jolla, CA). Statistical significance was considered if *p* ≤ 0.05.

## Figures and Tables

**Table 1:** This is a table. Tables should be placed in the main text near to the first time they are cited.

Primer Target	Primer Sequence (Forward)	Primer Sequence (Reverse)
GAPDH	5-CTGTGTCTTTCCGCTGTTTTC-3	5-TGTGCTGTGCTTATGGTCTCA-3
IL1β	5-AAAAATGCCTCGTGCTGTCT-3	5-TCGTTGCTTGTCTCTCCTTG-3
TNFα	5-AACTCCCAGAAAAGCAAGCA-3	5-CGAGCAGGAATGAGAAGAGG-3
iNOS	5-CTTGGAGCGAGTTGTGGATT-3	5-GGTGGGAGGGGTAGTGATG-3
IL-6	5-CTGCTCTGGTCTTCTGGAGT-3	5-GGTCTTGGTCCTTAGCCACT-3
Caspase3	5-CCATGTGTGAACTTGGTTGG-3	5-TCAACAATTTGAGGCTGCTG-3
CCL2	5-GCTGCTACTCATTCACTGGC-3	5-GGTGCTGAAGTCCTTAGGGT-3
IL10	5-CATCCGGGGTGACAATAA-3	5-TGTCCAGCTGGTCCTTCT-3
A3R	5-TTTACGGTCGGGAGTTCAAG-3	5-AGGGTTCATCATGGAGTTCG-3
A2aR	5-CCTCTTCTTCGCCTGTTTTG-3	5-GTTCCCGTCTTTCTGACTGC-3
TIMP1	5-GGTTCCCTGGCATAATCTGA-3	5-GTCATCGAGACCCCAAGGTA-3
TIMP2	5-CAAGTTCTTTGCCTGCATCA-3	5-GTTTCCAGGAAGGGATGTCA-3
IFNγ	5-GAACTGGCAAAAGGACGGTA-3	5-GGATCTGTGGGTTGTTCACC-3
COX2	5-CTGAGGGGTTACCACTTCCA-3	5-TGAGCAAGTCCGTGTTCAAG-3
MMP2	5-ACACTGGGACCTGTCACTCC-3	5-ACACGGCATCAATCTTTTCC-3
MMP9	5-CACTGTAACTGGGGGCAACT-3	5-CACTTCTTGTCAGCGTCGAA-3
Arg1	5-GGGAAGACACCAGAGGAGGT-3	5-TGATGCCCCAGATGACTTTT-3
IL12	5-CATCTGCTGCTCCACAAGAA-3	5-GAGACTCAGGGGAACTGCTG-3
IL4	5-TCCTTACGGCAACAAGGAAC-3	5-GTGAGTTCAGACCGCTGACA-3
MMP9	5-CACTGTAACTGGGGGCAACT-3	5-CACTTCTTGTCAGCGTCGAA-3

## Data Availability

Not applicable.
